# Neural correlates of cognitive dysfunction in fibromyalgia patients: Reduced brain electrical activity during the execution of a cognitive control task

**DOI:** 10.1016/j.nicl.2019.101817

**Published:** 2019-04-08

**Authors:** N. Samartin-Veiga, A.J. González-Villar, M.T. Carrillo-de-la-Peña

**Affiliations:** a Departamento de Psicoloxía Clínica e Psicobioloxía, Facultade de Psicoloxía, Universidade de Santiago de Compostela, Santiago de Compostela, Spain; b Psychological Neuroscience Lab, Research Center in Psychology, School of Psychology, University of Minho, Braga, Portugal

**Keywords:** Fibromyalgia, Event-related potentials (ERPs), Multi-Source Interference Task (MSIT), Cognitive control, Chronic pain, Attention, Dyscognition

## Abstract

**Objectives:**

Fibromyalgia (FM) is a generalized chronic pain syndrome of unknown aetiology. Although FM patients frequently complain of cognitive dysfunction, this is one of the least studied symptoms. Research on brain activity associated with the perceived cognitive impairment is particularly scarce. To address this gap, we recorded the brain electrical activity in participants during a cognitive control task.

**Methods:**

Electroencephalograms (EEGs) were recorded in 19 FM patients and 22 healthy controls (all women) while they performed the Multi-Source Interference Task (MSIT). We analyzed the amplitude of the frontal N2 and parietal P3 components elicited in control and interference trials and their relation with reaction times. We also explored the relationship of perceived cognitive dysfunction, assessed using visual analogue scales (VAS) and the Memory Failures of Everyday (MFE-30) test, with N2 and P3 amplitudes.

**Results:**

The N2 amplitudes were smaller in FM patients than in controls and were negatively associated with cognitive complaints. Unlike patients, healthy controls showed significant differences in the amplitude of P3 obtained from control vs. interference trials of the MSIT. Smaller N2 and P3 amplitudes were associated to longer reaction times.

**Conclusions:**

The findings suggest a reduction in frontal brain activity during performance of an interference task, which was associated with the patients' cognitive complaints. Findings on P3 suggest altered modulation of attention according to the task demands in FM patients. Deficits in flexibility in the allocation of attentional resources and cognitive control during complex tasks may explain the dyscognition reported by chronic pain patients.

## Highlights

• Fibromyalgia patients showed reduced N2 amplitude during the MSIT.
• P3 results suggests a difficulty in modulating the attentional resources in FM.
• Smaller N2 and P3 amplitudes were associated with longer reaction times.
• The decrease of N2 during the MSIT is associated with cognitive complaints.
• Attentional flexibility or cognitive control tasks are useful to assess FM symptoms.

## 1 Introduction

Fibromyalgia (FM) is a chronic pain syndrome of unknown aetiology and complex, variable evolution. This syndrome has an estimated prevalence in developed countries of between 1% and 4%, mainly affecting women (Alegre Martín, 2008). FM is characterized by widespread pain, constant tiredness and non-restorative sleep, but one of its most distinctive, and also rarely examined, symptoms is cognitive dysfunction. Although cognitive impairment has been reported in the 50% of the FM patients (Katz et al., 2004), the neural basis of such alterations is not clear.

Several studies have investigated the incidence of cognitive dysfunction in chronic pain syndromes, particularly in FM (Glass and Park, 2001). Most research efforts have focused on attention and memory processes, in which FM patients rather consistently show poorer performance than healthy controls (Grace et al., 1999; Glass and Park, 2001; Park et al., 2001; Dick et al., 2002; Dick et al., 2008; Cherry et al., 2009; Berryman et al., 2013; Landrø et al., 2013; Gelonch et al., 2016). Nevertheless, some reports failed to confirm cognitive deficits in FM patients performing neuropsychological tests of attention and memory (Suhr, 2003; Walitt et al., 2008; Glass et al., 2011; Walitt et al., 2016). Executive functions, including processes such as planning, attention, cognitive flexibility, inhibition or decision making, have been much less studied in FM patients. In 2014, Berryman et al. conducted a meta-analysis of 22 studies and reported that in patients with chronic pain there is a small impairment of executive function evaluated in a complex way, a result that the authors explain by the large variability of the tests used in the studies (Berryman et al., 2014). In view of the inconsistencies, some authors argue that the cognitive complaints made by FM patients are disproportionate to the actual performance displayed (Grace et al., 1999; Suhr, 2003; Castel et al., 2008; Schmidt-Wilcke et al., 2010) or may be explained by depression symptoms (Gelonch et al., 2016; Gelonch et al., 2017; Ojeda et al., 2017).

Regarding cognitive dysfunction and chronic pain, it has been proposed that pain captures attention and consumes part of the patient's attentional resources, thus impairing cognitive functioning (Eccleston and Crombez, 1999; Landrø et al., 2013; Tesio et al., 2015). This explanation is supported by neuroimaging studies that have assessed brain activity associated with higher cognitive processes, such as executive functioning in FM patients. Glass et al. (2011) observed lower activation in areas related to both pain perception and executive functioning in FM patients performing a Go-No Go task. Similarly, Seo et al. (2012) reported hypoactivation in the dorsolateral prefrontal cortex (DLPFC), the ventrolateral prefrontal cortex and the inferior parietal cortex in FM patients performing an n-back working memory task. Moreover, Schmidt-Wilcke et al. (2014) reported an increase in the activity of the anterior and middle cingulate cortex during the Go-No Go task in patients with reduced pain levels. These authors suggested that pain reduction may favour the availability of neural resources for other functions, such as inhibition. According to this concept, a hyperactive pain system, brought about by ongoing pain perception/anticipation/vigilance, takes up neural resources which are then no longer available for other networks and/or leads to poor integration of other brain networks in the pain system, and subsequently to their malfunctioning (Schmidt-Wilcke et al., 2014).

Although the analysis of brain electrical activity of FM patients while they perform cognitive tasks may be useful to clarify the existence of deficits in cognitive control and/or reduced allocation of attentional resources during executive functioning, studies conducted so far have used simple tasks such as the oddball paradigm. Using this task, it has been found that the amplitude of the N2 and P3 components of event-related potentials (ERPs) is smaller in FM patients than in controls (Ozgocmen et al., 2003; Yoldas et al., 2003; Alanoğlu et al., 2005). The smaller N2 amplitude has been interpreted as a deficit in executive control (Nieuwenhuis et al., 2003), including conflict and error monitoring and the ability to cancel a prepared response (Botvinick et al., 2004). On the other hand, the smaller P3 amplitude has been interpreted as an indicator of a reduced amount of attentional resources devoted to task execution (Folstein and Van Petten, 2008). Although these results seem to be consistent with attention deficit in FM, studies analyzing EEGs recorded during tasks that specifically assess processes such as executive control are scarce.

The overall objective of this study was to clarify the neural basis of dyscognition in FM by comparing the EEGs recorded in FM patients and healthy controls while they performed the Multi-Source Interference Task (MSIT), a task that activates the cognitive/attentional network. The MSIT, originally designed to elicit a robust interference effect in neuroimaging studies (Bush et al., 2003), has been widely used to study interference control in the healthy population and in numerous clinical conditions (Bush et al., 2003; Bush and Shin, 2006; Bush et al., 2008; Shehzad et al., 2012,; Veldhuijzen et al., 2012; Bush et al., 2013; Mao et al., 2014; Huerta-Albarrán et al., 2015; González-Villar et al., 2017). Previous fMRI studies in healthy individuals have shown that the MSIT activates the dorsal anterior midcingulate cortex, the DLPFC, premotor areas and parietal regions (Bush et al., 2003). These areas correspond to the cingulate-fronto-parietal network, which plays a critical role in attention and cognitive processing (Bush and Shin, 2006). In this study, we examined the N2 and P3 components of the ERPs and put them in relation to objective indices of performance (reaction times) and subjective cognitive complaints. We hypothesized that patients will show poorer MSIT performance along with smaller N2 and P3 amplitudes. Moreover, we expected to observe a negative relationship between subjective cognitive complaints and the N2 and P3 amplitudes.

## 2 Materials and methods

Nineteen patients with FM (mean age 43.58 ± 7.52) and twenty-two healthy controls (HCs) (mean age 45.14 ± 7.26), all right-handed, participated in the study. One FM participant was excluded due to inadequate performance of the task. The data included here are part of a larger study, and the patients' characteristics and behavioural results on task performance have already been described (González-Villar et al., 2017). The groups were matched by sex (all women), age and years of education (see Table 1). All FM patients had a previous diagnosis according to the ACR (1990) criteria by a primary care physician or rheumatologist, and had no other disease that could explain generalized pain. Furthermore, all patients included in the study reported moderate (often present) or severe (constantly present, life-disturbing) cognitive problems in the Symptom Severity Score of the Fibromyalgia Survey Questionnaire (Wolfe et al., 2011). Exclusion criteria for the FM group were the presence of neurological and psychiatric diseases (except depression or anxiety, since these two disorders show a high comorbidity with FM: see Aguglia et al., 2011), or history of substance abuse. The same exclusion criteria were applied to the control participants, who in addition, should not suffer from chronic pain problems.

**Table 1:** Descriptive statistics of the scales used and demographic variables, for fibromyalgia patients (FM) and healthy controls (HC).

Variable	FM (n = 18) mean (SD)	HC (n = 22) mean (SD)	t	p	Cohen's d
Age	43.9 (7.6)	45.1 (7.2)	−0.5	p = 0.61	–
Years of education	10.4 (3.5)	11.3 (3.3)	−0.8	p = 0.42	–
Symptom Severity Scale (SSS)	9.8 (2.3)	2.5(2.3)	10.0	p < 0.001	3.17
Widespread Pain Index (WPI)	14.0 (4.8)	1.7 (1.8)	10.2	p < 0.001	3.39
VAS. Pain	7.7 (2.0)	1.4 (1.6)	11.0	p < 0.001	3.47
VAS. Health status	7.6 (2.3)	2.3 (2.7)	6.7	p < 0.001	2.11
VAS. Mood status	7.4 (2.9)	2.9 (2.3)	5.4	p < 0.001	1.71
VAS. Functional affectation	8.2 (2.4)	0.1 (0.3)	15.4	p < 0.001	4.73
VAS. Non-restorative sleep	8.6 (2.1)	2.1 (2.4)	8.4	p < 0.001	2.88
VAS. Attention	6.9 (1.7)	2.2 (2.0)	7.9	p < 0.001	2.53
VAS. Memory	8.0 (1.8)	2.6 (2.1)	8.5	p < 0.001	2.76
VAS. Concentration	7.6 (1.9)	2.5 (2.4)	7.3	p < 0.001	2.35
Memory failures of everyday (MFE-30)	73.2 (22.5)	20.6(13.9)	8.9	p < 0.001	2.81

The participants were advised not to smoke or consume coffee, alcohol or other non-prescribed drugs before the evaluation; however, they were not asked to change their consumption of medically prescribed treatments. All participants had normal or corrected vision. Each participant was paid 25 euros to cover travel expenses.

The experimental procedure was approved by the research ethics committee of the University of Santiago de Compostela and all the participants were informed about the experiment and have signed informed consent before participation.

### 2.1 Clinical assessment

#### 2.1.1 Fibromyalgia symptoms assessment

##### 2.1.1.1 Fibromyalgia survey Questionnaire

We used the Spanish version of the Fibromyalgia Survey Questionnaire (FSQ) (Carrillo-de-la-Peña et al., 2015) originally published by Wolfe et al. (2011) and based on the diagnostic criteria proposed by Wolfe et al. (2010) with a self-report format. It includes a Symptom Severity Scale (SSS), which considers three key symptoms in FM: fatigue, cognitive problems (attention, concentration or memory) and non-restorative sleep. The FSQ also encompasses other symptoms such as abdominal pain, depression and headache. It also includes a Widespread Pain Index (WPI) indicating the number of body areas with pain reported by the patient.

##### 2.1.1.2 Visual-analogue scales

We used a series of Visual-Analogue Scales (VAS), which were created ad-hoc to evaluate the clinical status of participants. The scales consist of a set of horizontal 10 cm-long lines along which participants were asked to indicate their status for the following variables: pain, health status, mood, interference of their health in daily activities and non-restorative sleep. Also, we used VAS to assess subjective complaints regarding memory, attention and concentration during the last month. All scales were presented so that the right end indicated the worst condition and the left, the best.

##### 2.1.1.3 Memory failures of everyday (MFE-30) test

To assess memory problems, we used the Spanish validation of the modified Memory Failures of Everyday questionnaire (MFE-30) (Lozoya-Delgado et al., 2012) originally reported by Sunderland et al. (1984). The MFE-30, which evaluates memory lapses in everyday life, consists of a 30-items questionnaire with Likert responses (0: never or almost never, 1: rarely; 2: sometimes yes and sometimes not, 3: often, 4: always or almost always). In addition to subjective memory problems, the questionnaire evaluates other cognitive complaints related to perception, linguistics and praxical process.

## 3 Procedure and task

The questionnaires described above were first administered to the participants. For the EEG recording session, the participants sat in a comfortable chair in a dimly lit room isolated from external sounds. Each participant was fitted with an electrode cap and conductive paste was applied at each electrode to achieve the desired impedances.

**Fig. 1:**
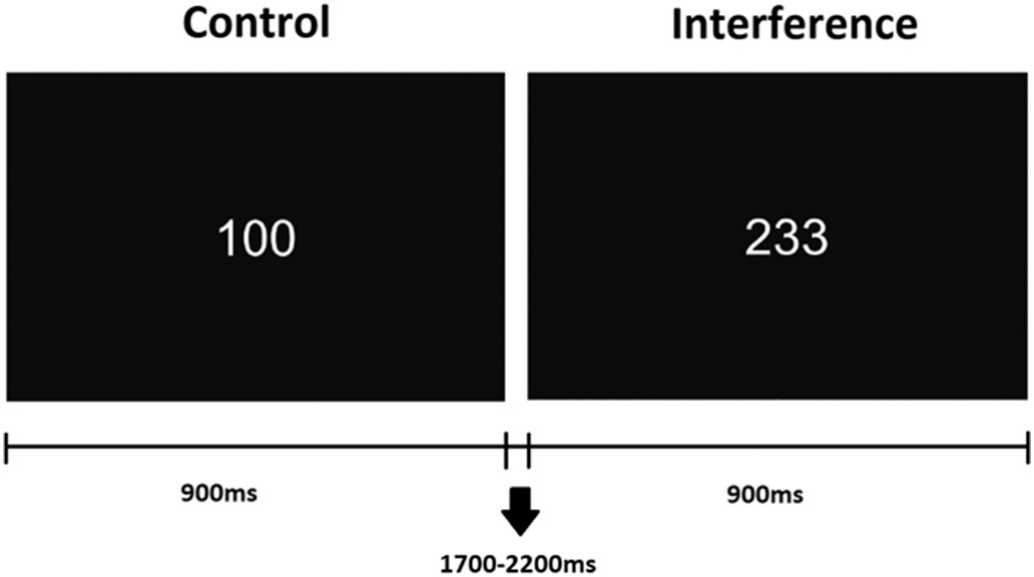
MSIT task. The subjects had to respond to the different number in the series, with their index (1), middle (2) or ring (3) fingers. During the control trials, the distractor stimuli were zeros (0) and the target number was placed in the position congruent with its position on the response box where the participant should respond. During interference trials, distractors were other numbers (1, 2 or 3) and the target was never placed in a position congruent with the position in the response box. In the first example the correct answer would be to press the “1” button with the index finger; and in the second example the correct answer would be to press the “2” button with the middle finger.

During the task, the participants were asked to look steadily at a point in front of them and to avoid moving their eyes and muscles. The MSIT instructions were clearly explained and all participants conducted 10 practice trials before performing the task. Participants had to respond via a response box with the index (1), middle (2) or ring (3) finger of their dominant hand to the different number of a series of three numbers displayed on the screen; they should respond to the identity of the different number and avoid responding to its position (see Fig. 1). In the control condition, the position of the different number was congruent with the response finger and was accompanied by zeros (i.e., “100”, “020”, “003”). In the interference condition, the position of the different number was incongruent with the response finger and was accompanied by two different numbers from 1 to 3 (i.e., “221”, “212”, “331”, “313”, “112”, “211”, “332”, “233”, “131”, “311”, “232” and “322”). The proportion of trials was 50% for each condition (50% control trials; 50% interference trials). The trials were presented randomly, although there were no more than three consecutive trials of the same condition or with the same response finger. Each stimulus was presented for 900 ms, with a random inter-stimulus interval (ISI) between 1700 and 2200 ms. Responses were recorded in the interval 150 to 2500 ms after the start of stimulus presentation. A total of 400 stimuli were presented to each participant. The task was designed and presented using PsychoPy (Peirce, 2009), on a 17-in. screen located 80 cm from the subject (See Fig. 1).

### 3.1 EEG recording and processing

The EEGs were recorded using the actiCHamp system (Brain Products Inc.), via 62 electrodes placed according to the 10–20 International System. The reference electrode was placed at the tip of the nose and the ground in the FPz position. Four additional surface electrodes were placed 1 cm above and below of the right eye and in the lateral canthus of both eyes to monitor vertical and horizontal ocular movements. Electrode impedances were kept below 10 KΩ. The EEG was digitized at 500 Hz and filtered with a 0.1–100 Hz on-line band pass filter and a 50 Hz Notch filter.

The EEG data were analyzed using the EEGlab v.3.13 toolbox (Delorme and Makeig, 2004). The EEGs were re-referenced to an average reference. EEG segments with artefacts were manually rejected after visual inspection. Data were digitally filtered using a 0.5 Hz high pass filter and a 30 Hz low-pass filter. We extracted epochs from 800 ms pre-stimulus to 1800 ms post-stimulus. An Independent Component Analysis (ICA) algorithm was used to remove the components associated with ocular or muscular activity. In three participants, one electrode had to be removed due to high levels of noise, and then reconstructed using spherical-spline interpolation. To obtain the ERPs, correctly responded epochs were averaged separately for control and interference trials, and baseline correction was applied from −200 to 0 ms. We selected two Regions of Interest (ROI) to measure N2 and P3 ERPs. For each participant, the N2 amplitude was computed as the mean voltage from 250 to 320 ms at the electrodes FC1, FCz, FC2 and Cz; while P3 amplitude was computed as the mean voltage from 350 to 600 ms at the electrodes P1, Pz, P2 and POz. These latency windows and electrodes were selected because they show the highest modulation of the N2 and P3 components.

### 3.2 Data analysis

The FM and HC groups were compared in relation to socio-demographic values, SSS, WPI, VAS, MFE data and behavioural results (reaction times and hits) using *t*-tests for independent samples. Repeated-measures ANOVAs were conducted for N2 and P3 amplitudes, with Condition (control vs. interference) as the within-subject factor and Group (FM vs. HC) as the between-subject factor. When the sphericity assumption was not fulfilled, Greenhouse-Geisser correction was used to determine the significance levels. When the ANOVA indicated that a factor was significant, t-tests with the Bonferroni correction were used in the post-hoc comparisons.

We also performed linear regression to analyze to what extent N2 and P3 amplitudes predicted reactions times, considering separately control and interference trials. Effect size analyses were undertaken to assess the magnitude of differences between groups in clinical variables. Specifically, Cohen's d was computed for t-tests, partial eta-squared [η_p_^2^] for ANOVAs, and Cohen's ƒ^2^ for linear regressions. Finally, Pearson's correlations were computed, only for the sample of FM patients, to evaluate associations between N2 and P3 amplitude and cognitive complaints (MFE-30 and VAS for memory, attention and concentration). All statistical analyses were performed with SPSS v. 20 (SPSS Inc., Chicago, IL, USA).

## 4 Results

Demographic and behavioural data were reported in a previous paper (González-Villar et al., 2017). There were no differences between groups in terms of age or education level. The results of the clinical assessment showed significant differences (p < 0.001) within FM patients and HCs in all the variables considered, with higher scores for patients with FM. These significative differences showed a Cohen's d superior to 0.80 for all the clinical measures, indicating a large effect size for the clinical variables (See Table 1). Regarding the behavioural performance of the MSIT, the reaction times (RT) were significantly slower in FM patients than in healthy controls. There were no group differences for the proportion of correct responses. Effect size analyses for the RT showed a large effect and the hit's effect size analyses for the control condition showed a medium effect, however for the interference condition showed a small effect (see Table 2).

**Table 2:** Means, SD (Standard Deviations), and t-tests of the behavioural data: RTs (Reaction Times, in milliseconds) and hits (percentage) for control and interference trials of the Multi-Source Interference Task (MSIT).

		FM mean (SD)	HC mean (SD)	t	p	Cohen's d
RT	Control	809.34 (149.12)	681.92 (122.92)	2.96	p < 0.01	0.93
	interference	1116.45 (206.34)	946.40 (144.08)	3.06	p < 0.01	0.96
Hits	Control	97.40% (3.37)	99.05% (1.83)	−1.85	p = 0.07	0.61
	Interference	87.19% (16.30)	91.59% (6.91)	−1.14	p = 0.25	0.35

For the N2 amplitude, the ANOVA revealed significant main effects for Group (F_(1,38)_ = 4.34; p = 0.04, η_p_^2^ = 0.10) and Condition (F_(1,38)_ = 6.23; p = 0.02, η_p_^2^ = 0.14); these effect sizes are considered as medium and large, respectively. The amplitude of this component was smaller in FM patients, and larger for the control than for the interference condition. The Group x Condition interaction was not significant (F_(1,38)_ = 0.50 p = 0.48, η_p_^2^ = 0.01).

**Fig. 2:**
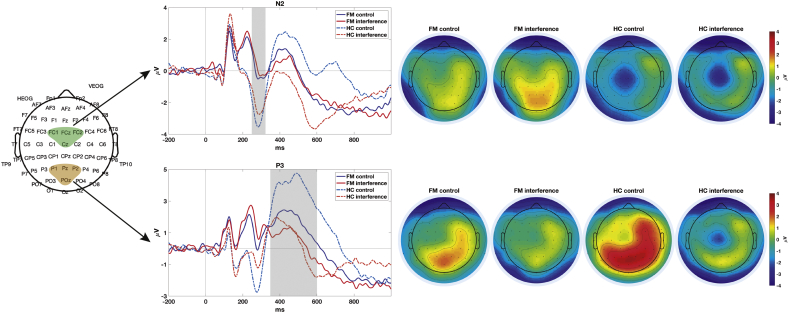
Left- Regions of Interest (ROIs) used to measure N2 (FC1, FCz, FC2 and Cz electrodes) and P3 (P1, Pz, P2 and POz). Center- Event related potentials obtained for the Fibromyalgia (FM) and Healthy Control (HC) groups in control and interference trials of the MSIT. The shaded areas show the time windows used to measure the mean amplitude of the ERPs. Right- The scalp distributions of the components for both groups and conditions.

For the P3 amplitude, the ANOVA showed significant effects for Condition (F_(1,38)_ = 29.64; p < 0.001, η_p_^2^ = 0.44) and for the Group x Condition interaction (F_(1,38)_ = 7.21; p = 0.01, η_p_^2^ = 0.16); both effect sizes are large. Post-hoc comparisons showed significantly larger P3 amplitudes to control than interference trials for the HCs (p < 0.001), while the difference was not significant for the patients (p = 0.071). (See Table 3, Fig. 2).

**Table 3:** Mean N2 and P3 amplitudes and SD (Standard Deviations).

	FM		HCs	
	Control	Interference	Control	Interference
Mean N2 amplitude (SD)	−0.07 (4.03)	0.28 (4.29)	−2.83 (3.85)	−2.21 (3.92)
Mean P3 amplitude (SD)	1.71 (5.53)	0.59 (6.58)	3.87 5.57)	0.57 (5.66)

To analyze the association between ERPs amplitudes and reaction times we performed linear regression analyses. The results show that the amplitude of N2 and P3 at control and interference conditions significantly predicted the reaction times of the participants (See Table 4). The effect size analysis for the linear regression of reaction times in the control condition showed a large effect (ƒ^2^ = 0.57), while for the interference condition it showed a medium effect (ƒ^2^ = 0.16).

**Table 4:** Linear regression results (standardized beta coefficient) for Reaction Times (RT; dependent variable) using N2 and P3 amplitude in both conditions of the task (control and interference) as independent variables.

Linear regression results	RT Control condition β(p)	RT Interference condition β(p)
N2	0.463 (p = 0.001)	0.481 (p = 0.010)
P3	−0.570 (p < 0.001)	−0.415 (p = 0.025)

In FM patients, N2 amplitude was significantly correlated with attention, memory and concentration (assessed by VAS). Smaller N2 amplitudes were associated with larger self-perceived cognitive dysfunction (See Table 5).

**Table 5:** Pearson r correlations of the Visual-Analogue Scales (VAS) and Memory Failures of Everyday (MFE-30) score, with N2 and P3 obtained in MSIT control and interference trials, for FM patients.

	VAS Attention r (p)	VAS memory r (p)	VAS concentration r (p)	MFE-30 r (p)
N2 control	0.620 (p = 0.006)	0.613 (p = 0.007)	0.744 (p < 0.001)	0.361 (p = 0.141)
N2 interference	0.541 (p = 0.021)	0.566 (p = 0.014)	0.696 (p = 0.001)	0.298 (p = 0.230)
P3 control	0.147 (p = 0.560)	−0.091 (0.718)	0.093 (p = 0.714)	−0.210 (p = 0.402)
P3 interference	0.188 (p = 0.454)	0.015 (p = 0.954)	0.224 (p = 0.371)	−0.109 (p = 0.667)

## 5 Discussion

The main aim of this study was to understand the neural mechanisms involved in the cognitive dysfunction reported by FM patients. To this end, we recorded EEGs in FM patients and healthy controls while they performed the Multi-Source Interference Task (MSIT), a test designed to explore processes related to attentional control and interference processing. We examined the frontal N2 and parietal P3 components of the ERPs and correlated them with indices of subjective cognitive complaints.

According to our hypotheses concerning neural activity related to the performance of the MSIT, we found a decrease in N2 amplitude in FM patients. The N2 component has been interpreted as a neural correlate of conflict detection and monitoring, necessary for the response program updating and successful inhibition (Huster et al., 2013). Thus, the smaller N2 amplitudes found in the patients may indicate their lower involvement in the processing of the task stimuli. This finding is in line with previous research on spontaneous brain activity and EEG time-frequency analyses in FM patients. Several studies have shown altered patterns of spontaneous brain activity in the patients (like increased connectivity between the default mode network and other cortical structures), suggestive of a dominance of endogenous top-down influences that may be limiting the processing of incoming stimuli (Ceko et al., 2015; Ichesco et al., 2014). Using time-frequency analyses, we observed lower theta activity in patients than in controls during the performance of the MSIT (González-Villar et al., 2017). As the theta frequency band has been related to N2 and is also considered an indicator of conflict monitoring and response competition (Müller and Anokhin, 2012), the similar results found using different markers of brain activity lend greater robustness to our findings.

The N2 wave has a fronto-central scalp distribution and its neural basis has been located in the dorsal portion of the anterior cingulate cortex (ACC), a brain structure involved in monitoring the concurrent and competitive coactivation of responses or stimuli representations (Van Veen and Carter, 2002; Botvinick et al., 2004). A previous neuroimaging study reported less activation of the ACC in chronic pain patients while they were performing the MSIT, suggesting that this region plays a major role in the cognitive dysfunction (Mao et al., 2014). Thus, our results are also consistent with previous neuroimaging findings obtained using the MSIT with FM and chronic pain patients.

Against our expectations and contrary to previous findings using the oddball task (Ozgocmen et al., 2003; Yoldas et al., 2003; Alanoğlu et al., 2005), we were not able to confirm a general reduction in the amplitude of the P3 component in FM patients. We used a specific task to assess cognitive control and observed different patterns of brain electrical activity across trials: healthy controls showed larger P3 amplitude in control than interference trials, while the patients did not show significant modulation. The smaller P3 amplitudes in the interference condition may be explained by its increased difficulty, as compared to the control one. Given that the temporo-parietal component P3 has been considered an index of attentional processing of the target stimulus (Polich, 2007), our findings suggest that FM patients may not use appropriate strategies to solve the task, assigning similar amounts of attentional resources to stimuli processing in both, control and interference conditions.

Altogether, the decrease in the amplitude of frontal N2 and the lack of difference in the P3 amplitude across trials suggest that patients with FM may have difficulty in modulating attention according to the task demands. This pattern of neural activity may explain the poorer behavioural performance of the patients in the MSIT; in fact, smaller N2 and P3 amplitudes successfully predicted longer reaction times. Previous studies have also reported slower reactions times and larger number of errors in the patients (Veldhuijzen et al., 2012; González-Villar et al., 2017).

In this study we attempted to relate the brain activity recorded during the MSIT to the cognitive symptoms reported by the patients. There is some discussion regarding the nature of cognitive complaints by chronic pain patients and whether they are disproportionate or objective (Grace et al., 1999; Gervais et al., 2001; Suhr, 2003; Walitt et al., 2016; Gelonch et al., 2017). With the aim of obtaining a more accurate measure of cognitive symptoms, we used both Visual-Analogue Scales (VAS) and the Memory Failures of Everyday questionnaire (MFE-30). Considering the cognitive profile of the patients included in the study, our evaluations confirmed the presence of a moderate to severe dysfunction in attention, memory and concentration in the FM group. According to our hypothesis, in FM patients, we found significant correlations between N2 amplitude and complaints about attention, memory and concentration, with smaller amplitudes indicating greater cognitive dysfunction. These results suggest a link between a poor involvement in the task (i.e. reduced monitoring of stimuli) and greater subjective impression of dyscognition. In line with our results, Walitt et al. (2016) reported that subjective complaints of FM patients correlate with a decrease in BOLD response (in supramarginal gyrus/primary somatosensory cortex, posterior insula, left inferior temporal cortex, left inferior parietal cortex and bilateral occipital cortex) during the execution of a working memory task (n-back). Moreover, other studies have also reported correlations between the self-reported complaints and objective performance indices of cognition capacity (Park et al., 2001; Leavitt and Katz, 2006; Verdejo-García et al., 2009; Landrø et al., 2013; Tesio et al., 2015).

The above findings can be interpreted following the hypothesis that cognitive processes- such as attention, inhibition or cognitive control, and pain perception depend on overlapping networks (Eccleston and Crombez, 1999; Landrø et al., 2013; Tesio et al., 2015). It is known that both painful stimuli and cognitive tasks (n-back, MSIT, go/no-Go, etc) robustly activate regions in the prefrontal cortex and superior parietal lobes, as well as the ACC, mid-cingulate cortex (MCC), supplementary motor area, and the anterior insular cortex (Schmidt-Wilcke et al., 2014). In chronic pain patients, the constant processing of pain may use up neural resources that may be necessary to perform tasks that require attentional load, thus affecting cognitive processing (Eccleston and Crombez, 1999; Glass et al., 2011; Seo et al., 2012; Schmidt-Wilcke et al., 2014). Nevertheless, there is some controversy about this idea, because the interactions between pain and cognition are poorly defined. Some researchers have observed poorer performance of patients with FM in neuropsychological tests of attention and memory (Glass and Park, 2001; Park et al., 2001; Dick et al., 2002, Dick et al., 2008; Landrø et al., 2013; Tesio et al., 2015; Gelonch et al., 2016; Ojeda et al., 2017) and cognitive control tasks (Verdejo-García et al., 2009; Cherry et al., 2012; Berryman et al., 2013; Gelonch et al., 2016), while others have not (Suhr, 2003; Glass et al., 2011; Walitt et al., 2016). The discrepancies between studies may stem from the different nature of the tests used to assess cognitive abilities, differences in the samples and other methodological issues.

We acknowledge some limitations of our study. First, the medication taken by the patients may have affected the results. Although participants were instructed to not consume more medication than necessary, for ethical reasons we did not asked them to discontinue prescribed medication. Many of the patients were taking anxiolytic and antidepressant drugs that may affect the Central Nervous System, and therefore the electrophysiological indices. In addition, some participants reported suffering insomnia and distress, which may also affect the findings. Furthermore, it should be noted that we used a version of the MSIT (random presentation of control and interference trials) that deviates from the standard presentation in blocks, and that may also have affected the results obtained. The inclusion of simple control trials between the more difficult interference trials may partly explain the higher amplitude of P3 in the control condition, a result that is at odds with our previous report (González-Villar et al., 2017). Moreover, the sample of this study was exclusively composed of women. Although FM is a syndrome suffered mostly by female (Wolfe et al., 1995), this aspect presents a limitation in our study in terms of its generalization to the male population. Future research should also establish whether the susceptibility of FM patients to suffer from cognitive deficits can be explained by the presence of other symptoms such as pain or depression.

In conclusion, FM patients and healthy controls were significantly different in brain electrical activity indices obtained during the MSIT that were related to the behavioural performance of the task. The data on the P3 component suggests that patients may have difficulty in modulating the number of attentional resources needed to perform activities with varying cognitive loads. We also found that the subjective cognitive dysfunction reported by the patients is related to an objective, neural index (N2 amplitude). Taken together, the N2 and P3 results allow us to better understand the cognitive dysfunction reported by FM patients: a poor stimuli monitoring may lead to less cognitive flexibility and worse performance in tasks that require cognitive control. Simple tasks may not be sensitive enough to bring out the patient's dyscognition, thus explaining some of the inconsistencies in previous literature. Besides, these findings provide evidence in favour of the hypothesis of some overlap between networks involved in pain and executive function, thus helping to explain why patients with chronic pain may develop cognitive problems. Finally, the results provide knowledge that may improve the diagnosis and treatment of these patients: the use of brain electrical activity biomarkers could help to characterize the patients' dyscognition and stablish profiles to design more individualized treatment strategies.

## Financial support

This study was supported by funding from from the Spanish Government (Ministerio de Economía y Competitividad; grant number PSI2013–45818-R] and from the Galician Government (Consellería de Cultura, Educación e Ordenación Universitaria; axudas para a consolidación e Estruturación de unidades de investigación competitivas do Sistema universitario de Galicia; grant number GPC 2014/047). A.G.V. was supported by a grant from Xunta de Galicia (Axudas de apoio á etapa de formación posdoutoral 2018).

## References

[bb0005] Aguglia A., Salvi V., Maina G., Rossetto I., Aguglia E. (2011). Fibromyalgia syndrome and depressive symptoms: comorbidity and clinical correlates. J. Affect. Disord..

[bb0010] Alanoğlu E., Ulaş U.H., Özdağ F., Odabaşı Z., Çakçı A., Vural O. (2005). Auditory event-related brain potentials in fibromyalgia syndrome. Rheumatol. Int..

[bb0015] Alegre Martín J. (2008). La Fibromialgia es una entidad primaria del dolor: ¿hay deterioro cognitivo en la Fibromialgia?. Rev. Soc. Esp. Dolor.

[bb0020] Berryman C., Stanton T.R., Bowering K.J., Tabor A., McFarlane A., Moseley G.L. (2013). Evidence for working memory deficits in chronic pain: a systematic review and meta-analysis. Pain.

[bb0025] Berryman C., Stanton T.R., Bowering K.J., Tabor A., McFarlane A., Moseley G.L. (2014). Do people with chronic pain have impaired executive function? a meta-analytical review. Clin. Psychol. Rev..

[bb0030] Botvinick M.M., Cohen J.D., Carter C.S. (2004). Conflict monitoring and anterior cingulate cortex: an update. Trends Cogn. Sci..

[bb0035] Bush G., Shin L.M. (2006). The multi-source interference task: an fMRI task that reliably activates the cingulo-frontal-parietal cognitive/attention network. Nat. Proto. Electron. Ed..

[bb0040] Bush G., Shin L.M., Holmes J., Rosen B.R., Vogt B.A. (2003). The multi-source interference task: validation study with fMRI in individual subjects. Mol. Psychiatry.

[bb0045] Bush G., Spencer T.J., Holmes J., Shin L.M., Valera E.M., Seidman L.J., Biederman J. (2008). Functional magnetic resonance imaging of methylphenidate and placebo in attention-deficit/hyperactivity disorder during the multi-source interference task. Arch. Gen. Psychiatry.

[bb0050] Bush G., Holmes J., Shin L.M., Surman C., Makris N., Mick E., Biederman J. (2013). Atomoxetine increases fronto-parietal functional MRI activation in attention-deficit/hyperactivity disorder: a pilot study. Psychiatry Res. Neuroimaging.

[bb0055] Carrillo-de-la-Peña M.T., Triñanes Y., González-Villar A., Romero-Yuste S., Gómez-Perretta C., Arias M., Wolfe F. (2015). Convergence between the 1990 and 2010 ACR diagnostic criteria and validation of the Spanish version of the fibromyalgia survey questionnaire (FSQ). Rheumatol. Int..

[bb0060] Castel A., Cascón R., Salvat M., Sala J., Padrol A., Pérez M., Rull M. (2008). Rendimiento cognitivo y percepción de problemas de memoria en pacientes con dolor crónico: con fibromialgia versus sin fibromialgia. Rev. Soc. Esp. Dolor.

[bb0065] Ceko M., Gracely J.L., Fitzcharles M.A., Seminowicz D.A., Schweinhardt P., Bushnell M.C. (2015). Is a responsive default mode network required for successful working memory task performance?. J. Neurosci..

[bb0070] Cherry B.J., Weiss J., Barakat B.K., Rutledge D.N., Jones C.J. (2009). Physical performance as a predictor of attention and processing speed in fibromyalgia. Arch. Phys. Med. Rehabil..

[bb0075] Cherry B.J., Zettel-Watson L., Shimizu R., Roberson I., Rutledge D.N., Jones C.J. (2012). Cognitive performance in women aged 50 years and older with and without fibromyalgia. J. Gerontol. B Psychol. Sci. Soc. Sci..

[bb0080] Delorme A., Makeig S. (2004). EEGLAB: an open source toolbox for analysis of single-trial EEG dynamics including independent component analysis. J. Neurosci. Methods.

[bb0085] Dick B., Eccleston C., Crombez G. (2002). Attentional functioning in fibromyalgia, rheumatoid arthritis, and musculoskeletal pain patients. Arthritis Care Res..

[bb0090] Dick B.D., Verrier M.J., Harker K.T., Rashiq S. (2008). Disruption of cognitive function in fibromyalgia syndrome. Pain.

[bb0095] Eccleston C., Crombez G. (1999). Pain demands attention: a cognitive–affective model of the interruptive function of pain. Psychol. Bull..

[bb0100] Folstein J.R., Van Petten C. (2008). Influence of cognitive control and mismatch on the N2 component of the ERP: a review. Psychophysiology.

[bb0105] Gelonch O., Garolera M., Valls J., Rosselló L., Pifarré J. (2016). Executive function in fibromyalgia: comparing subjective and objective measures. Compr. Psychiatry.

[bb0110] Gelonch O., Garolera M., Valls J., Rosselló L., Pifarré J. (2017). Cognitive complaints in women with fibromyalgia: are they due to depression or to objective cognitive dysfunction?. J. Clin. Exp. Neuropsychol..

[bb0115] Gervais R.O., Russell A.S., Green P., Allen L.M., Ferrari R., Pieschl S.D. (2001). Effort testing in patients with fibromyalgia and disability incentives. J. Rheumatol..

[bb0120] Glass J.M., Park D.C. (2001). Cognitive dysfunction in fibromyalgia. Curr. Rheumatol. Rep..

[bb0125] Glass J.M., Williams D.A., Fernandez-Sanchez M.L., Kairys A., Barjola P., Heitzeg M.M., Schmidt-Wilcke T. (2011). Executive function in chronic pain patients and healthy controls: different cortical activation during response inhibition in fibromyalgia. J. Pain.

[bb0130] González-Villar A.J., Samartin-Veiga N., Arias M., Carrillo-de-la-Peña M.T. (2017). Increased neural noise and impaired brain synchcastelronization in fibromyalgia patients during cognitive interference. Sci. Rep..

[bb0135] Grace G.M., Nielson W.R., Hopkins M., Berg M.A. (1999). Concentration and memory deficits in patients with fibromyalgia syndrome. J. Clin. Exp. Neuropsychol..

[bb0140] Huerta-Albarrán R., Poblano A., Santana-Vargas D., Castro-Sierra E., Haro R., Garza-Morales S. (2015). Error related negativity and multi-source interference task in children with attention deficit hyperactivity disorder-combined type. Arq. Neuropsiquiatr..

[bb0145] Huster R.J., Enriquez-Geppert S., Lavallee C.F., Falkenstein M., Herrmann C.S. (2013). Electroencephalography of response inhibition tasks: functional networks and cognitive contributions. Int. J. Psychophysiol..

[bb0150] Ichesco E., Schmidt-Wilcke T., Bhavsar R., Clauw D.J., Peltier S.J., Kim J., Harris R.E. (2014). Altered resting state connectivity of the insular cortex in individuals with fibromyalgia. J. Pain.

[bb0155] Katz R.S., Heard A.R., Mills M., Leavitt F. (2004). The prevalence and clinical impact of reported cognitive difficulties (fibrofog) in patients with rheumatic disease with and without fibromyalgia. JCR.

[bb0160] Landrø N.I., Fors E.A., Våpenstad L.L., Holthe Ø., Stiles T.C., Borchgrevink P.C. (2013). The extent of neurocognitive dysfunction in a multidisciplinary pain centre population. is there a relation between reported and tested neuropsychological functioning?. Pain.

[bb0165] Leavitt F., Katz R.S. (2006). Distraction as a key determinant of impaired memory in patients with fibromyalgia. J. Rheumatol..

[bb0170] Lozoya-Delgado P., Ruiz-Sánchez de León J.M., Pedrero-Pérez E.J. (2012). Validación de un cuestionario de quejas cognitivas para adultos jóvenes: relación entre las quejas subjetivas de memoria, la sintomatología prefrontal y el estrés percibido. Rev. Neurol..

[bb0175] Mao C.P., Zhang Q.L., Bao F.X., Liao X., Yang X.L., Zhang M. (2014). Decreased activation of cingulo-frontal-parietal cognitive/attention network during an attention-demanding task in patients with chronic low back pain. Neuroradiology.

[bb0180] Müller V., Anokhin A.P. (2012). Neural synchrony during response production and inhibition. PLoS ONE.

[bb0185] Nieuwenhuis S., Yeung N., Van Den Wildenberg W., Ridderinkhof K.R. (2003). Electrophysiological correlates of anterior cingulate function in a go/no-go task: effects of response conflict and trial type frequency. Cogn. Affect. Behav. Neurosci..

[bb0190] Ojeda B., Dueñas M., Salazar A., Mico J.A., Torres L.M., Failde I. (2017). Factors influencing cognitive impairment in neuropathic and musculoskeletal pain and fibromyalgia. Pain Med..

[bb0195] Ozgocmen S., Yoldas T., Kamanli A., Yildizhan H., Yigiter R., Ardicoglu O. (2003). Auditory P300 event related potentials and serotonin reuptake inhibitor treatment in patients with fibromyalgia. Ann. Rheum. Dis..

[bb0200] Park D.C., Glass J.M., Minear M., Crofford L.J. (2001). Cognitive function in fibromyalgia patients. Arthritis Rheum..

[bb0205] Peirce J.W. (2009). Generating stimuli for neuroscience using PsychoPy. Front. Neuroinformatics.

[bb0210] Polich J. (2007). Updating P300: an integrative theory of P3a and P3b. Clin. Neurophysiol..

[bb0215] Schmidt-Wilcke T., Wood P., Lürding R. (2010). Cognitive impairment in patients suffering from fibromyalgia. an underestimated problem. Schmerz (Berlin, Germany).

[bb0220] Schmidt-Wilcke T., Kairys A., Ichesco E., Fernandez-Sanchez M.L., Barjola P., Heitzeg M., Williams D.A. (2014). Changes in clinical pain in fibromyalgia patients correlate with changes in brain activation in the cingulate cortex in a response inhibition task. Pain Med..

[bb0225] Seo J., Kim S.H., Kim Y.T., Song H.J., Lee J.J., Kim S.H., Lee S.J. (2012). Working memory impairment in fibromyalgia patients associated with altered frontoparietal memory network. PLoS ONE.

[bb0230] Shehzad Z., DeYoung C.G., Kang Y., Grigorenko E.L., Gray J.R. (2012). Interaction of COMT val 158 met and externalizing behavior: relation to prefrontal brain activity and behavioral performance. NeuroImage.

[bb0235] Suhr J.A. (2003). Neuropsychological impairment in fibromyalgia: relation to depression, fatigue, and pain. J. Psychosom. Res..

[bb0240] Sunderland A., Harris J.E., Gleave J. (1984). Memory failures in everyday life following severe head injury. J. Clin. Exp. Neuropsychol..

[bb0245] Tesio V., Torta D.M., Colonna F., Leombruni P., Ghiggia A., Fusaro E., Castelli L. (2015). Are fibromyalgia patients cognitively impaired? objective and subjective neuropsychological evidence. Arthritis Care Res..

[bb0250] Van Veen V., Carter C.S. (2002). The anterior cingulate as a conflict monitor: fMRI and ERP studies. Physiol. Behav..

[bb0255] Veldhuijzen D.S., Sondaal S.F., Oosterman J.M. (2012). Intact cognitive inhibition in patients with fibromyalgia but evidence of declined processing speed. J. Pain.

[bb0260] Verdejo-García A., López-Torrecillas F., Calandre E.P., Delgado-Rodríguez A., Bechara A. (2009). Executive function and decision-making in women with fibromyalgia. Arch. Clin. Neuropsychol..

[bb0265] Walitt B., Roebuck-Spencer T., Bleiberg J., Foster G., Weinstein A. (2008). Automated neuropsychiatric measurements of information processing in fibromyalgia. Rheumatol. Int..

[bb0270] Walitt B., Čeko M., Khatiwada M., Gracely J.L., Rayhan R., VanMeter J.W., Gracely R.H. (2016). Characterizing “fibrofog”: subjective appraisal, objective performance, and task-related brain activity during a working memory task. NeuroImage.

[bb0275] Wolfe F., Ross K., Anderson J., Russell I.J., Hebert L. (1995). The prevalence and characteristics of fibromyalgia in the general population. Arthritis Rheum..

[bb0280] Wolfe F., Clauw D.J., Fitzcharles M.A., Goldenberg D.L., Katz R.S., Mease P., Yunus M.B. (2010). The American College of Rheumatology preliminary diagnostic criteria for fibromyalgia and measurement of symptom severity. Arthritis Care Res..

[bb0285] Wolfe F., Clauw D.J., Fitzcharles M.A., Goldenberg D.L., Häuser W., Katz R.S., Winfield J.B. (2011). Fibromyalgia criteria and severity scales for clinical and epidemiological studies: a modification of the ACR preliminary diagnostic criteria for fibromyalgia. J. Rheumatol..

[bb0290] Yoldas T., Ozgocmen S., Yildizhan H., Yigiter R., Ulvi H., Ardicoglu O. (2003). Auditory p300 event-related potentials in fibromyalgia patients. Yonsei Med. J..

